# Correction to: The heat shock factor 20-HSF4-cellulose synthase A2 module regulates heat stress tolerance in maize

**DOI:** 10.1093/plcell/koaf173

**Published:** 2025-08-05

This is a correction to: Ze Li, Zerui Li, Yulong Ji, Chunyu Wang, Shufang Wang, Yiting Shi, Jie Le, Mei Zhang, The heat shock factor 20-HSF4-cellulose synthase A2 module regulates heat stress tolerance in maize, *The Plant Cell*, Volume 36, Issue 7, July 2024, Pages 2652–2667, https://doi.org/10.1093/plcell/koae106

In the originally published version of the manuscript diacritics were missed on the third co-author Yulong Ji to indicate equal contribution to the article. This has now been added and the Author Note emended respectively.

These emendations have been made to the article.

